# Association of the glucose metabolism continuum (fasting plasma glucose/HbA1c) with tear-film stability and secretion: ocular surface evidence across non-diabetes, prediabetes, and diabetes

**DOI:** 10.3389/fendo.2026.1788051

**Published:** 2026-04-13

**Authors:** Huan Xiang, Zengsheng Zhou

**Affiliations:** 1 Department of Ophthalmology, First People’s Hospital of Wuyi County, Jinhua, Zhejiang, China; 2 Department of Anesthesiology, Maternal and Child Health Hospital of Wuyi County, Jinhua, Zhejiang, China

**Keywords:** diabetes, dry eye disease, fasting plasma glucose, HbA1c, non-invasive tear break-up time, prediabetes, Schirmer test

## Abstract

**Background:**

Whether gradations in glycemia across non-diabetes, prediabetes, and diabetes relate to tear−film homeostasis and dry eye disease (DED) remains uncertain, and explicitgraded association evidence is limited.

**Methods:**

In a single−center cross−sectional study (April 2024–March 2025), we enrolled 300 adults by quota (*n* = 100 per stratum). Same−day hemoglobin A1c (HbA1c) and fasting plasma glucose (FPG) classified strata and were also modeled continuously. Co−primary outcomes were non-invasive tear break−up time (NIBUT) and Schirmer I (no anesthesia). Secondary outcomes included fluorescein tear break-up time (TBUT), ocular surface disease index (OSDI), National Eye Institute (NEI) staining, meiboscore, tear meniscus height, bulbar redness, tear osmolarity, and matrix metalloproteinase-9 (MMP−9). DED was defined as OSDI ≥13 plus ≥1 sign [NIBUT <10 s, osmolarity ≥308 mOsm/L or intereye difference ≥8 mOsm/L, or NEI staining score ≥2 (beyond trace)]. Multivariable analysis of covariance (ANCOVA) and logistic regression adjusted for prespecified demographic, clinical, and environmental covariates. Restricted cubic splines tested non-linearity, and Benjamini–Hochberg false discovery rate (FDR) controlled multiplicity for secondary endpoints.

**Results:**

Mean NIBUT was 11.8, 10.5, and 9.2 s, and Schirmer I was 13.8, 12.1, and 10.4 mm across non-diabetes, prediabetes, and diabetes (both *p*−trend < 0.001). Adjusted differences versus non-diabetes were −1.20 and −2.50 s for NIBUT and −1.60 and −3.10 mm for Schirmer (all *p* ≤ 0.006). Per 1% higher HbA1c, NIBUT decreased by 0.72 s and Schirmer by 1.15 mm, and DED odds increased (aOR 1.31; all *p* < 0.001) with no significant non-linearity. DED proportion within strata was 24.0%, 34.0%, and 51.0% (prediabetes aOR 1.60, *p* = 0.049; diabetes aOR 2.90, *p* = 0.001). Osmolarity abnormality and MMP−9 positivity rose across strata and with HbA1c, remaining significant after FDR control. Using FPG instead of HbA1c yielded concordant effects.

**Conclusions:**

Higher glycemic status, including prediabetes, was associated with shorter NIBUT, lower Schirmer, and higher odds of DED and inflammatory signs, although the absolute between-stratum differences were modest in magnitude. Longitudinal studies are needed to assess the temporality and the practical relevance of these cross-sectional associations.

## Introduction

Dry eye disease (DED) is a chronic, multifactorial ocular surface disorder in which loss of tear-film homeostasis is central and symptoms often impair visual function and daily activities (1, 2). Patient-reported impact is routinely captured with validated instruments such as the Ocular Surface Disease Index (OSDI) (3). Beyond individual burden, DED imposes substantial economic costs from medical care and productivity loss (4). At the same time, dysglycemia along the non-diabetes to diabetes continuum is highly prevalent in the population, creating a broad exposure base by which systemic metabolic stressors could influence the ocular surface (5, 6). Together, these considerations motivate inquiry into whether gradations in glycemia correlate with measurable decrements in tear-film stability and volume and with a higher DED burden (1, 2).

Pathophysiologically, DED is sustained by a feed-forward loop in which tear hyperosmolarity and ocular surface inflammation amplify epithelial stress and neurosensory abnormalities, while chronic hyperglycemia may contribute to these processes (7). Elevated tear osmolarity, commonly operationalized as ≥308mOsm/L in either eye or an intereye difference ≥8mOsm/L, serves as a biomarker of homeostatic failure (8). Inflammatory activity is frequently indexed by the tear matrix metalloproteinase-9 (MMP-9) point-of-care test (9). Diabetes-related small-fiber and autonomic neuropathy, detectable with corneal confocal microscopy, can blunt reflex tearing and alter blink dynamics, reducing aqueous secretion and promoting evaporative loss (10). In parallel, meibomian gland dysfunction (MGD) compromises the lipid layer and increases evaporation (11). These mechanisms map cleanly onto testable outcomes, tear-film stability via breakup time [non-invasive break-up time (NIBUT) or fluorescein tear break-up time (TBUT)] and tear volume via Schirmer testing and tear meniscus height, within diagnostic guidance from the Tear Film and Surface Society Dry Eye Workshop II (TFOS DEWS II) (12).

Multiple systematic reviews and meta-analyses report that adults with diabetes, compared with non-diabetic peers, have shorter TBUT, lower Schirmer values, and a higher prevalence of DED (13–15). A large population-based work similarly links diabetes with increased DED risk and symptom burden and suggests that worse metabolic control tracks with more severe ocular surface findings (16). However, evidence in prediabetes and explicit dose–response modeling across the full glycemic continuum remains sparse (13, 15). Methodological heterogeneity also persists (12, 17). Addressing these gaps with standardized phenotyping, environmental measurement, and continuous modeling is needed to clarify thresholds and gradients from non-diabetes through prediabetes to diabetes (13, 16, 17).

Our primary aim was to quantify the association between glycemia and tear-film homeostasis across the non-diabetes to diabetes continuum using two complementary co-primary endpoints: NIBUT (tear-film stability) and Schirmer I without anesthesia (aqueous secretion). By interrogating prediabetes and modeling the full glycemic spectrum with complementary co-primary endpoints and environmentally standardized phenotyping, this study seeks to delineate cross-sectional exposure–response patterns that may inform hypothesis generation and the design of future longitudinal and interventional studies.

## Methods

### Study design

We conducted a single−center, prospective cross−sectional study at First People’s Hospital of Wuyi County (Zhejiang, China) from April 2024 to March 2025, to examine associations between glycemic status and tear−film homeostasis. The protocol prespecified two co−primary outcomes, non-invasive tear break−up time (NIBUT) and Schirmer I without anesthesia, and a dual exposure framework in which glycemia was modeled both categorically (non-diabetes, prediabetes, diabetes) and continuously [hemoglobin A1c (HbA1c), fasting plasma glucose (FPG)]. The protocol conformed to the Declaration of Helsinki and was approved by the Ethics Committee of the First People’s Hospital of Wuyi County. All participants provided written informed consent prior to any study procedures.

### Setting and participants

Adults attending comprehensive ophthalmology or diabetes/metabolic clinics were screened consecutively and enrolled into one of the three glycemic strata (non-diabetes, prediabetes, and diabetes) using predefined biomarker cut points (see below). Recruitment was quota-balanced (*n* = 100 per stratum) to ensure adequate precision for between-stratum contrasts. Once a stratum reached its cap, further eligible individuals in that stratum were not enrolled. Screening was performed by trained staff using a standardized checklist, and ocular surface findings were not used for eligibility beyond the prespecified exclusion criteria. Exclusion criteria included pregnancy/lactation, contact lens wear within 7 days, current use of topical ocular medications (other than artificial tears), ocular surgery/trauma within 6 months, active ocular infection or inflammation, Sjögren’s syndrome, or prespecified conditions/medications expected to invalidate tear testing (e.g., marked eyelid malposition, punctal occlusion, active allergic conjunctivitis, ocular surface burns/cicatrizing disease, systemic isotretinoin use within 6 months, or recent chemotherapy/head–neck radiotherapy). Common systemic medications with potential tear effects (e.g., anticholinergics, antihistamines, antidepressants, beta-blockers, diuretics) were not excluded but were recorded and adjusted. Because quota sampling was based on glycemic strata, reported proportions reflect this clinic-based, quota-sampled cohort and should not be interpreted as population prevalence. All participants were asked to refrain from artificial tears and eye makeup for ≥12 h before testing.

### Glycemic assays and classification

After an overnight fast, venous blood was collected for FPG (enzymatic hexokinase method in an accredited clinical laboratory) and HbA1c (high−performance liquid chromatography aligned to NGSP/DCCT). Same−day results were used to classify glycemic strata according to the American Diabetes Association thresholds: non-diabetes (HbA1c < 5.7% and FPG < 100 mg/dL), prediabetes (HbA1c 5.7%–6.4% or FPG 100–125 mg/dL), and diabetes (HbA1c ≥ 6.5% or FPG ≥ 126 mg/dL or known diabetes on therapy). In addition to strata, we analyzed HbA1c as a continuous exposure per 1% and FPG per 10 mg/dL to quantify exposure–response relationships.

### Ocular surface assessment and measurement order

All testing occurred before any clinical instillation, after a 10−min acclimation in a quiet room, in a fixed least−to−most invasive order to minimize test interference: Ocular Surface Disease Index (OSDI), non-contact imaging for NIBUT and infrared meibography using the Keratograph 5M (Oculus, Wetzlar, Germany, upper and lower lids scored 0–3 per lid and summed to a 0–6 meiboscore), calibrated anterior−segment imaging (Keratograph 5M) for tear meniscus height at the central lower lid and bulbar redness graded 0–4 against standardized images, point−of−care tear osmolarity (TearLab Osmolarity System, TearLab Corp, San Diego, USA) sampled from the inferior lateral meniscus (one reading per eye, repeated once if an error code occurred) with abnormality defined as ≥308 mOsm/L in either eye or intereye difference ≥8 mOsm/L, point−of−care MMP−9 qualitative lateral−flow assay (InflammaDry; QuidelOrtho, San Diego, CA, USA) read at 10 min by masked staff, fluorescein tear break−up time (TBUT) measured as the interval between last blink and first corneal dark spot after instillation of minimal fluorescein (fluorescein sodium strip lightly wetted with preservative-free saline; excess fluid shaken off; strip tip briefly touched to the inferior bulbar conjunctiva; estimated delivered volume ~1 µL), National Eye Institute (NEI) corneal/conjunctival staining (0–15 composite), and Schirmer I without anesthesia using standard strips for 5 min with eyes gently closed. For NIBUT, we recorded first−break times in triplicate per eye and averaged the three first−break values to obtain the eye−level measure. TBUT was also recorded three times per eye with minimal fluorescein volume. MMP−9 was recorded as positive if a visible red test line appeared with an intact blue control line at 10 min; two masked readers interpreted the assay, with adjudication of any discordant reads.

### Outcomes and case definitions

The two co−primary outcomes were NIBUT (seconds) representing tear−film stability and Schirmer I (millimeters) representing aqueous secretion; secondary outcomes included TBUT, OSDI score, NEI staining, meiboscore, tear meniscus height, bulbar redness, osmolarity abnormality, and MMP−9 positivity. DED was defined *a priori* as OSDI ≥13 plus at least one sign of homeostatic loss, namely, NIBUT <10 s, tear hyperosmolarity defined above, or epithelial staining beyond trace operationalized as an NEI staining score ≥2. This symptom plus sign composite follows the TFOS DEWS II diagnostic framework and reduces misclassification from symptom–sign discordance. In a prespecified robustness analysis used for historical comparability, we evaluated an alternative composite definition using a more stringent sign set: OSDI ≥13 plus TBUT <5 s or Schirmer ≤10 mm or NEI staining ≥5. For participant-level analyses, continuous eye-level measurements were averaged across eyes, whereas binary signs were considered positive if either eye met the criterion to capture unilateral disease; sensitivity analyses using worse-eye values for continuous measures and a both-eye requirement for binary outcomes were reported.

### Covariates and environmental controls

Potential confounders were prespecified and ascertained by interview and chart review, including age; sex with menopausal status recorded for women; body mass index; current smoking; tear−affecting systemic medications such as anticholinergics, antihistamines, antidepressants, and isotretinoin; thyroid disease; rheumatologic disease; and daily screen time. Because ambient conditions influence evaporation and stability, room temperature and relative humidity were measured at each visit with a calibrated thermohygrometer and entered as covariates. All ocular assessments were conducted by certified examiners masked to laboratory glycemia, and laboratory personnel were masked to ocular outcomes. For participants with known diabetes, duration since diagnosis and current antidiabetic treatment regimen were abstracted from records when available and were summarized descriptively; these variables were additionally included in prespecified sensitivity models restricted to the diabetes stratum. Chart-documented neuropathy, diabetic retinopathy, and nephropathy/albuminuria were also recorded when available for descriptive context only. Because ascertainment was incomplete and non-standardized, these variables were not included in the primary adjusted models.

### Sample size and power

With *n* = 100 per stratum and standard deviations of approximately 3.0 s for NIBUT and 5.0–5.5 mm for Schirmer I, we anticipated roughly 80% power at two−sided *α* = 0.05 to detect between−group differences of at least 1.2 s for NIBUT and 2.0 mm for Schirmer when comparing non-diabetes with either prediabetes or diabetes, with higher power for ordinal trend tests and continuous−exposure models across the full glycemic spectrum.

### Statistical analysis

Descriptive statistics are presented as unadjusted means with standard deviations and *t*−based 95% confidence intervals (CIs) for continuous variables and as percentages with Wilson 95% CIs for categorical variables. For the co−primary endpoints, we fit multivariable linear models (ANCOVA) regressing each outcome on glycemic stratum with non-diabetes as the reference and adjusted for prespecified covariates, including age, sex/menopausal status, body mass index, current smoking, tear−affecting medications, thyroid disease, rheumatologic disease, daily screen time, room temperature, and relative humidity. We report adjusted pairwise differences for prediabetes versus non-diabetes and diabetes versus nondiabetes with 95% CI and an ordinal *p*−for−trend. Secondary continuous outcomes used the same framework, while binary outcomes (DED prevalence, osmolarity abnormality, MMP−9 positivity, OSDI ≥ 13) used multivariable logistic regression to estimate adjusted odds ratios with 95% CIs and an ordinal trend test. Exposure–response was evaluated by modeling HbA1c per 1% and FPG per 10 mg/dL in separate adjusted models, and restricted cubic splines with four knots at the 5th, 35th, 65th, and 95th percentiles assessed departures from linearity via a Wald chi−square comparison to a linear term. Robust Huber–White standard errors were used throughout. For multiplicity, both co−primary endpoints were required to be significant at *α* = 0.05 (no FDR applied to co−primary tests); within families of secondary endpoints tabulated together, we applied Benjamini–Hochberg false discovery rate control at *q* = 0.05. For transparency, we summarized the analytic families, model-level hypotheses, displayed *p*-values/contrasts, and multiplicity handling across the reported primary, secondary, spline, and sensitivity analyses (**Supplementary Table 1**). Model assumptions were examined with residual diagnostics, influence statistics, and variance inflation factors, and logistic models were assessed for calibration. An overview of endpoint families, the number of tests, and the multiplicity approach was provided; HbA1c and FPG were modeled separately, given collinearity, and adjusted *R*^2^ was reported for the co−primary linear models.

#### Sensitivity and robustness analyses

Prespecified sensitivity analyses included substitution of FPG for HbA1c as the continuous exposure in otherwise identical models, an alternative DED definition using OSDI ≥13 plus TBUT <5 s or Schirmer ≤10 mm or NEI staining ≥5 to reflect more stringent fluorescein−based criteria, exclusion of participants taking tear−affecting systemic medications, and a reclassification of osmolarity abnormality that required an absolute value ≥308 mOsm/L without using the intereye difference criterion to evaluate robustness to Δ−based classification. Robustness analyses included restriction of the prediabetes stratum to participants meeting both HbA1c and FPG criteria, separate symptom-only and sign-only endpoint models (e.g., OSDI ≥13 alone and NIBUT <10 s, Schirmer ≤10 mm, or NEI staining ≥2 irrespective of symptoms), alternative eye−level aggregation (worse-eye values for continuous measures and a both−eye requirement for binary outcomes), and (among participants with diabetes) additional adjustment for diabetes duration and treatment regimen in models of HbA1c versus ocular outcomes.

#### Data quality and missing data

All devices underwent daily calibration according to manufacturer guidance, examiners completed prestudy interrater reliability exercises with periodic retraining, and the testing environment was standardized and recorded. Inclusion required same−day laboratory and ocular testing, yielding complete primary exposures and outcomes. Covariate missingness was trivial, and complete−case analysis was performed.

## Results

**Figure 1:**
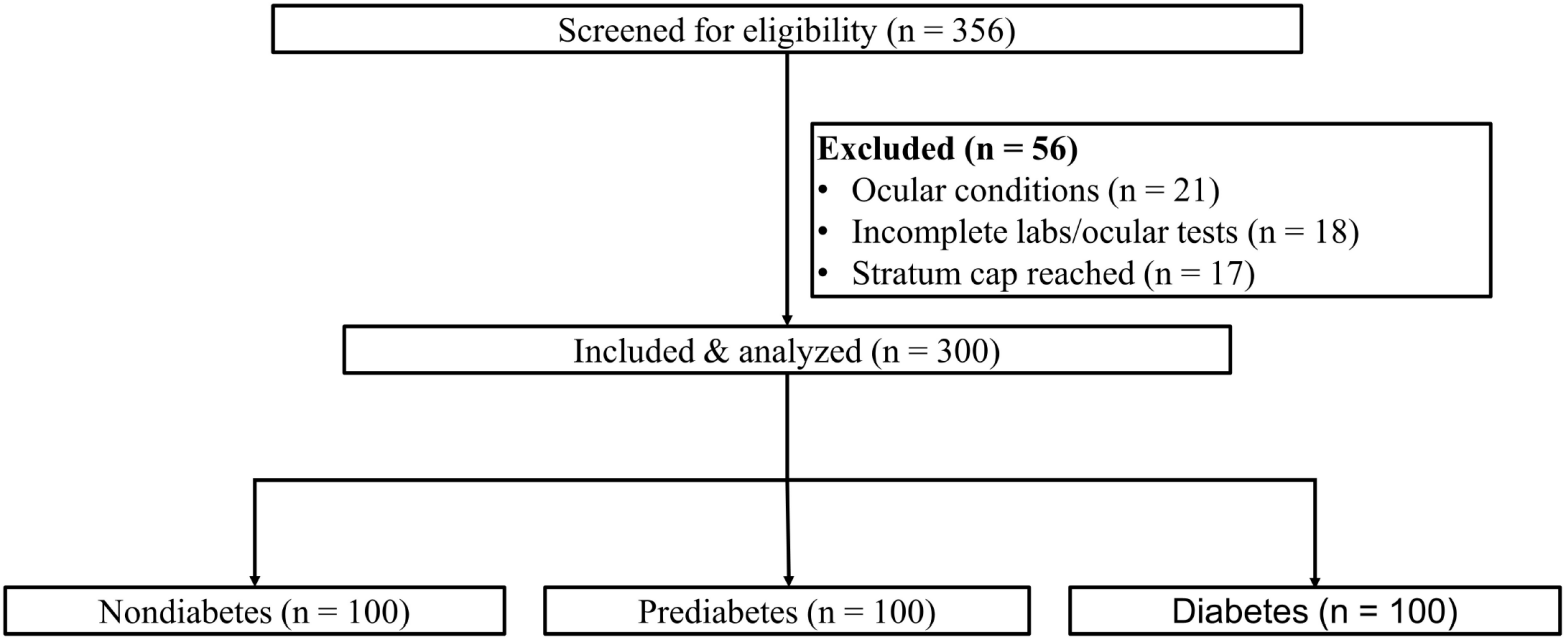
Participant flow diagram. “Stratum cap” indicates equal quota sampling to improve precision for between-group contrasts.

Of 356 individuals screened between April 2024 and March 2025, 56 were excluded (21 ocular exclusions, 18 incomplete same-day testing, 17 due to quota caps), yielding 300 participants with equal quotas across non-diabetes, prediabetes, and diabetes (*n* = 100 per group; **Figure 1**). The cohort’s mean age was 56.8 ± 11.2 years and 57.0% were women. Age, sex, and ambient testing conditions were similar across groups (all *p* > 0.05), whereas body mass index increased with higher glycemic status (25.7 ± 3.9, 27.1 ± 4.1, and 28.3 ± 4.3 kg/m^2^; *p* < 0.001). As expected, HbA1c and fasting plasma glucose differed by stratum (both *p* < 0.001). Other covariates, including smoking, tear-affecting medications, thyroid/rheumatologic disease, and screen time, were comparable (**Table 1**). Within the prediabetes stratum, 25 (25.0%) met HbA1c criteria only, 15 (15.0%) met FPG criteria only, and 60 (60.0%) met both criteria (**Supplementary Table 2**). Among participants in the diabetes stratum, median duration of diagnosed diabetes was 8.0 years [IQR 3.5–13.0], and the most common treatment regimens were metformin monotherapy (35.0%), other oral agents without insulin (37.0%), and insulin-containing therapy (20.0%) (**Supplementary Table 2**). Chart-documented complications in the diabetes stratum were diabetic retinopathy in 24.0%, nephropathy/albuminuria in 12.0%, and peripheral neuropathy in 18.0%; these chart-derived data were descriptive only and were not used as primary adjustment covariates because standardized phenotyping was unavailable (**Supplementary Table 2**).

**Table 1:** Baseline characteristics by glycemic stratum.

Characteristic	Overall (*N* = 300)	Non-diabetes (*n* = 100)	Prediabetes (*n* = 100)	Diabetes (*n* = 100)	*p* (global)
Age, years (mean±SD)	56.8±11.2	55.6±11.1	56.4±10.9	58.4±11.5	0.180
Female, %	57.0	58.0	55.0	58.0	0.860
BMI, kg/m^2^ (mean±SD)	27.0±4.2	25.7±3.9	27.1±4.1	28.3±4.3	<0.001
Current smoker, %	17.3	16.0	17.0	19.0	0.770
Tear-affecting meds, %	22.0	21.0	22.0	23.0	0.920
Thyroid or rheumatologic disease, %	9.3	8.0	10.0	10.0	0.830
Screen time, h/day (mean±SD)	6.1±2.4	6.0±2.3	6.1±2.4	6.2±2.5	0.820
Room temperature, °C (mean±SD)	22.5±1.2	22.5±1.2	22.4±1.2	22.6±1.3	0.620
Relative humidity,% (mean±SD)	43.7±4.9	43.5±5.0	43.8±4.8	43.9±5.0	0.790
HbA1c, % (mean±SD)	6.4±1.1	5.4±0.2	6.0±0.2	7.8±0.9	<0.001
FPG, mg/dL (mean±SD)	113±24	92±7	108±8	139±28	<0.001

**Figure 2:**
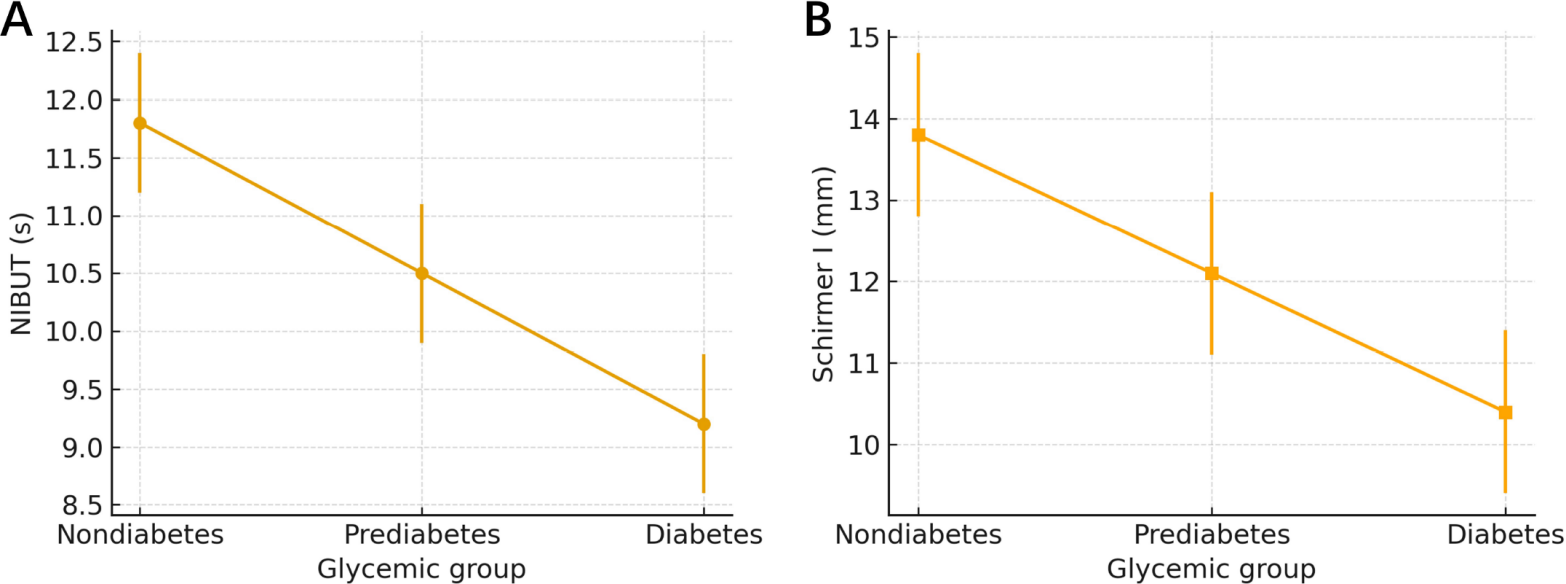
**(A)** NIBUT across strata. Adjusted contrasts (ANCOVA): pre vs. non Δ = −1.2s (*p*=0.003); DM vs. non Δ=−2.5s (*p*<0.001); *p*-trend < 0.001. Error bars are 95% CIs for group means. **(B)** Schirmer I across strata. Adjusted contrasts (ANCOVA): pre vs. non Δ = −1.6mm (*p* = 0.006); DM vs. non Δ = −3.1mm (p<0.001); *p*-trend < 0.001. Error bars are 95% CIs for group means.

Tear-film stability and aqueous secretion showed a graded pattern across glycemic strata (**Table 2**, **Figure 2**). Mean NIBUT was 11.8 s (95% CI 11.2–12.4) in non-diabetes, 10.5 s (9.9–11.1) in prediabetes, and 9.2 s (8.6–9.8) in diabetes. In adjusted models, the mean difference versus non-diabetes was −1.20 s (95% CI −1.90 to −0.40; *p* = 0.003) for prediabetes and −2.50 s (−3.30 to −1.70; *p* < 0.001), with a significant trend across strata (*p* < 0.001). Schirmer I values were 13.8 mm (12.8–14.8), 12.1 mm (11.1–13.1), and 10.4 mm (9.3–11.5); adjusted differences versus non-diabetes were −1.60 mm (95% CI −2.80 to −0.50; *p* = 0.006) and −3.10 mm (−4.40 to −1.80; *p* < 0.001), with *p*-trend <0.001.

**Table 2:** Ocular surface outcomes by glycemic stratum with adjusted comparisons.

A. Continuous outcomes (co-primary first)									
Outcome (units)	Non-diabetes, mean±SD [95%CI]	Prediabetes, mean±SD [95%CI]	Diabetes, mean±SD [95%CI]	Adj. mean difference (ANCOVA) vs. non-diabetes [95%CI]	*p* (pre)	Adj. mean difference (ANCOVA) vs. non-diabetes [95%CI]	*p* (DM)	*p*-trend	FDR *q*
NIBUT, s	11.8±3.1 [11.2–12.4]	10.5±3.2 [9.9–11.1]	9.2±3.3 [8.6–9.8]	−1.20 [−1.90, −0.40]	0.003	−2.50 [−3.30, −1.70]	<0.001	<0.001	–
SchirmerI, mm	13.8±5.2 [12.8–14.8]	12.1±5.3 [11.1–13.1]	10.4±5.5 [9.3–11.5]	−1.60 [−2.80, −0.50]	0.006	−3.10 [−4.40, −1.80]	<0.001	<0.001	–
TBUT, s	9.5±3.0 [8.9–10.1]	8.6±3.1 [8.0–9.2]	7.4±3.2 [6.8–8.0]	−0.80 [−1.40, −0.20]	0.01	−2.00 [−2.70, −1.30]	<0.001	<0.001	0.001
OSDI (0–100)	12.4±9.1 [10.6–14.2]	15.8±10.2 [13.8–17.8]	19.6±12.1 [17.3–21.9]	+3.10 [+0.70, +5.60]	0.012	+6.70 [+3.90, +9.50]	<0.001	<0.001	0.002
NEI staining (0–15)	2.8±2.1 [2.4–3.2]	3.6±2.4 [3.1–4.1]	4.7±2.8 [4.1–5.3]	+0.70 [+0.20, +1.20]	0.006	+1.70 [+1.00, +2.30]	<0.001	<0.001	0.003
Meiboscore (0–6)	1.2±1.0 [1.0–1.4]	1.5±1.1 [1.3–1.7]	1.8±1.2 [1.6–2.0]	+0.30 [+0.00, +0.60]	0.047	+0.60 [+0.30, +0.90]	<0.001	<0.001	0.012
Tear meniscus height,mm	0.22±0.06 [0.21–0.23]	0.20±0.06 [0.19–0.21]	0.18±0.06 [0.17–0.19]	−0.02 [−0.03, −0.01]	0.001	−0.04 [−0.05, −0.03]	<0.001	<0.001	0.001
Bulbar redness (0–4)	1.1±0.5 [1.0–1.2]	1.2±0.5 [1.1–1.3]	1.4±0.6 [1.3–1.5]	+0.10 [+0.00, +0.20]	0.041	+0.30 [+0.10, +0.40]	<0.001	<0.001	0.014
B. Binary outcomes									
Outcome	Non-diabetes, % [95%CI]	Prediabetes, % [95%CI]	Diabetes, % [95%CI]	aOR vs. non (pre) [95%CI]	*p* (pre)	aOR vs. non (DM) [95%CI]	*p* (DM)	*p*-trend	FDR *q*
DED (OSDI ≥ 13 + ≥1 sign)	24.0 [15.6–32.4]	34.0 [24.7–43.3]	51.0 [41.2–60.8]	1.60 [1.00, 2.60]	0.049	2.90 [1.50, 5.60]	0.001	<0.001	0.008
Osmolarity ≥ 308mOsm/L or Δ≥8mOsm/L	19.0 [11.3–26.8]	28.0 [19.2–36.8]	39.0 [29.4–48.6]	1.60 [1.00, 2.60]	0.046	2.50 [1.40, 4.50]	0.002	0.001	0.01
MMP-9 positive	17.0 [9.7–24.3]	24.0 [15.6–32.4]	36.0 [26.6–45.4]	1.50 [0.90, 2.50]	0.11	2.40 [1.30, 4.20]	0.004	0.002	0.015
OSDI ≥ 13	31.0 [21.9–40.1]	42.0 [32.2–51.8]	59.0 [49.2–68.8]	1.60 [1.00, 2.50]	0.05	3.20 [1.80, 5.70]	<0.001	<0.001	0.006

Secondary continuous outcomes moved concordantly with the co-primary endpoints and remained significant after multiplicity control (**Table 2**). Fluorescein TBUT decreased (9.5 ± 3.0, 8.6 ± 3.1, 7.4 ± 3.2 s; adjusted *p*-trend < 0.001; FDR *q* = 0.001). OSDI symptom scores increased (12.4 ± 9.1, 15.8 ± 10.2, 19.6 ± 12.1; *q* = 0.002). NEI staining worsened (2.8 ± 2.1, 3.6 ± 2.4, 4.7 ± 2.8; *q* = 0.003) and meiboscore increased (1.2 ± 1.0, 1.5 ± 1.1, 1.8 ± 1.2; *q* = 0.012). Tear meniscus height was lower with greater dysglycemia (0.22 ± 0.06, 0.20 ± 0.06, 0.18 ± 0.06 mm; *q* = 0.001), and bulbar redness scores were higher (1.1 ± 0.5, 1.2 ± 0.5, 1.4 ± 0.6; *q* = 0.014). All effects persisted after adjustment for prespecified covariates.

**Figure 3:**
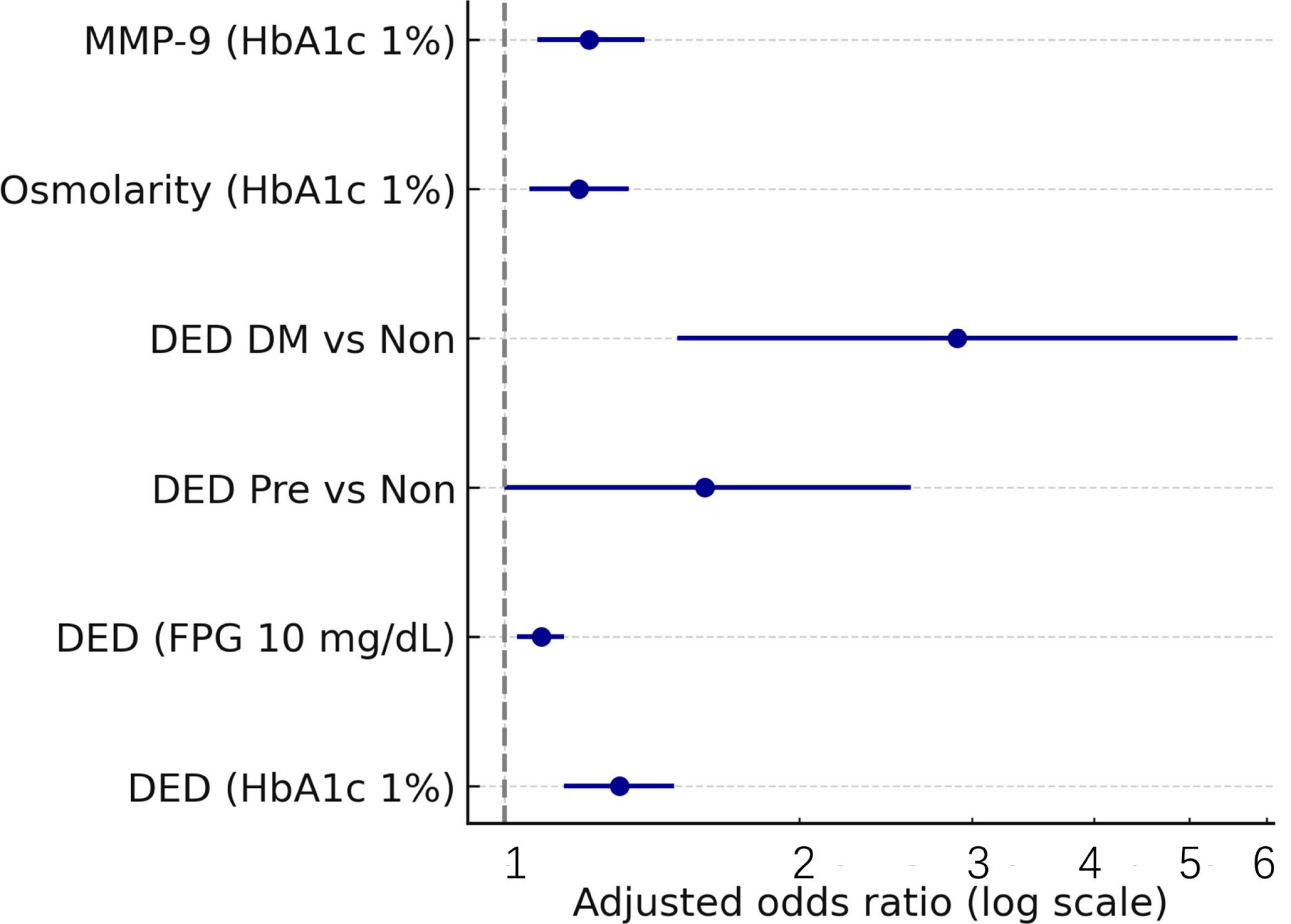
Association of glycemia with DED and inflammatory signs (forest plot). Adjusted odds ratios (95% CI): DED per 1% HbA1c 1.31 (1.15–1.49), *p* < 0.001; DED per 10mg/dL FPG 1.09 (1.03–1.15), *p* = 0.003; DED prediabetes vs. non-diabetes 1.60 (1.00–2.60), *p* = 0.049; DED DM vs. non 2.90 (1.50–5.60), *p* = 0.001; osmolarity abnormal per 1% HbA1c 1.19 (1.06–1.34), *p* = 0.004; MMP-9 positive per 1% HbA1c 1.22 (1.08–1.39), *p* = 0.002.

Using the prespecified composite definition (OSDI ≥ 13 plus ≥ 1 sign), the proportion meeting dry eye disease criteria within the quota-sample strata was 24.0% (95% CI 15.6–32.4) in non-diabetes, 34.0% (24.7–43.3) in prediabetes, and 51.0% (41.2–60.8) in diabetes (**Table 2**, **Figure 3**). Adjusted odds ratios versus non-diabetes were 1.60 (95% CI 1.00–2.60; *p* = 0.049) for prediabetes and 2.90 (1.50–5.60; *p* = 0.001), with *p*-trend <0.001 and FDR *q* = 0.008. Tear-osmolarity abnormality (≥308 mOsm/L in either eye or intereye difference ≥8 mOsm/L) increased from 19.0% to 28.0% to 39.0%, corresponding to adjusted odds ratios of 1.60 (95% CI 1.00–2.60; *p* = 0.046) and 2.50 (1.40–4.50; *p* = 0.002; *p*-trend = 0.001; *q* = 0.010). MMP-9 positivity rose from 17.0% to 24.0% to 36.0%, with a significant diabetes versus non-diabetes contrast (aOR 2.40; *p* = 0.004), a non-significant prediabetes versus non-diabetes contrast (*p* = 0.11), and *p*-trend = 0.002 (*q* = 0.015). The proportion with OSDI ≥13 alone increased from 31.0% to 42.0% to 59.0% with adjusted odds ratios of 1.60 (95% CI 1.00–2.50; *p* = 0.050) and 3.20 (1.80–5.70; *p* < 0.001; *p*-trend < 0.001; *q* = 0.006). Because recruitment was quota-balanced by glycemic strata, these proportions should be interpreted as within-sample estimates rather than population prevalence. Objective sign-only endpoints (e.g., NIBUT < 10 s, Schirmer ≤ 10 mm, and NEI staining ≥ 2 irrespective of symptoms) also increased across strata (**Supplementary Table 3**).

**Figure 4:**
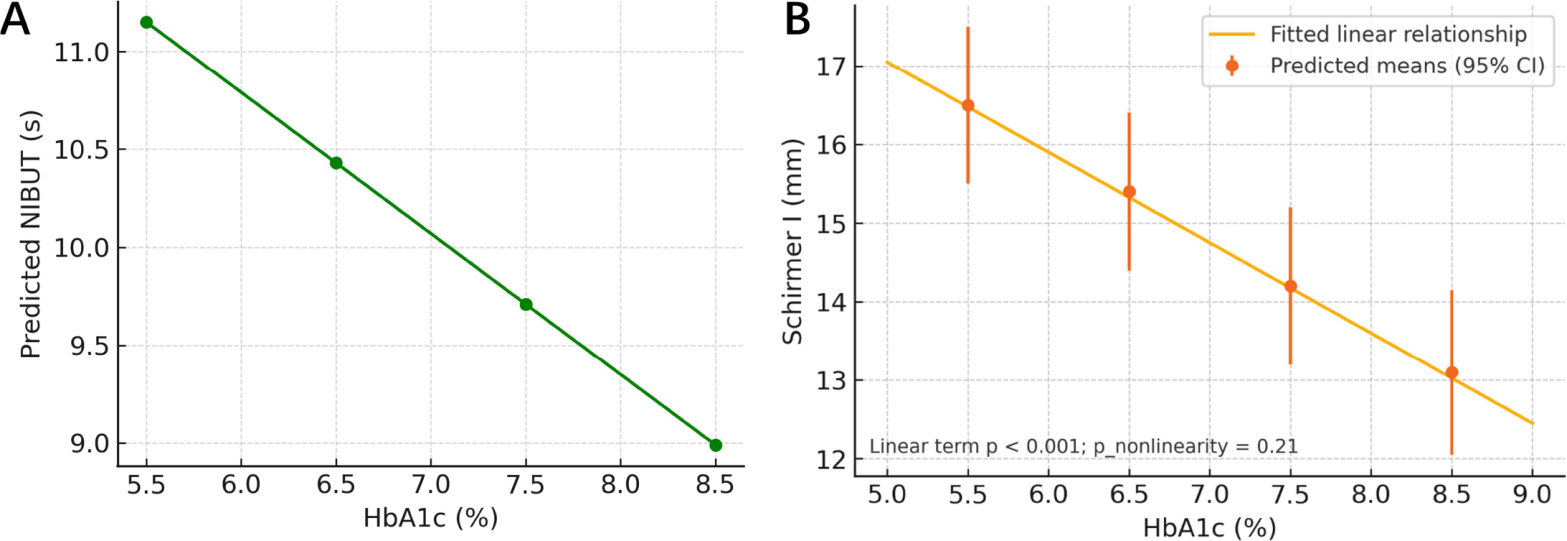
**(A)** HbA1c vs. NIBUT (exposure–response). Restricted cubic splines found no significant non-linearity, so the fitted line shows the linear effect (*β* = −0.72s per 1% HbA1c; *p* < 0.001); points show predicted means with 95% CIs at HbA1c 5.5%, 6.5%, 7.5%, and 8.5%; p_non-linearity=0.17. **(B)** HbA1c vs. Schirmer I (exposure–response). Linear effect shown (*β* = −1.15 mm per 1% HbA1c; *p* < 0.001); predicted means with 95% CIs at 5.5%, 6.5%, 7.5%, and 8.5%; p_non-linearity = 0.21.

Higher HbA1c was associated with shorter NIBUT and lower Schirmer I after adjustment (**Table 3**, **Figure 4**). Each 1% increase in HbA1c corresponded to a −0.72 s difference in NIBUT (95% CI −1.02 to −0.42; *p* < 0.001) and a −1.15 mm difference in Schirmer I (95% CI −1.75 to −0.56; *p* < 0.001). The adjusted odds of DED increased by a factor of 1.31 per 1% higher HbA1c (95% CI 1.15–1.49; *p* < 0.001). Restricted cubic spline tests detected no significant non-linearity for HbA1c with either NIBUT or Schirmer (*p* = 0.17 and *p* = 0.21, respectively). Using FPG in place of HbA1c yielded concordant effects: −0.18 s for NIBUT and −0.28 mm for Schirmer per 10 mg/dL higher FPG (both *p* ≤ 0.004), with DED odds ratio 1.09 per 10 mg/dL (95% CI 1.03–1.15; *p* = 0.003). Osmolarity abnormality and MMP-9 positivity also increased with higher HbA1c (aOR 1.19 and 1.22 per 1% HbA1c; *p* = 0.004 and *p* = 0.002, respectively), and all secondary continuous models remained significant after FDR control (*q* ≤ 0.009).

**Table 3:** Multivariable models (primary and key secondary endpoints).

Endpoint	Exposure	Effect (*β* or aOR) [95%CI]	*p*	FDR *q*
NIBUT (s)	HbA1c per 1%	*β* = −0.72 [−1.02, −0.42]	<0.001	−
SchirmerI (mm)	HbA1c per 1%	*β* = −1.15 [−1.75, −0.56]	<0.001	−
TBUT (s)	HbA1c per 1%	*β* = −0.65 [−0.95, −0.34]	<0.001	<0.001
DED (composite)	HbA1c per 1%	aOR = 1.31 [1.15, 1.49]	<0.001	<0.001
Osmolarity abnormal	HbA1c per 1%	aOR = 1.19 [1.06, 1.34]	0.004	0.009
MMP-9 positive	HbA1c per 1%	aOR = 1.22 [1.08, 1.39]	0.002	0.006
NIBUT (s)	FPG per 10mg/dL	*β* = −0.18 [−0.28, −0.08]	<0.001	<0.001
Schirmer I (mm)	FPG per 10mg/dL	*β* = −0.28 [−0.48, −0.09]	0.004	0.009
DED (composite)	FPG per 10mg/dL	aOR = 1.09 [1.03, 1.15]	0.003	0.006
DED (alt. definition)	HbA1c per 1%	aOR = 1.27 [1.09, 1.49]	0.002	0.006
Non-linearity (RCS)	HbA1c → NIBUT	*χ*² (*df* = 2), *p* = 0.170	–	–
Non-linearity (RCS)	HbA1c → Schirmer	*χ*² (*df* = 2), *p* = 0.210	–	–

Findings were robust across prespecified sensitivity analyses (**Supplementary Table 3**, **Table 3**). When DED was redefined using the alternative composite incorporating fluorescein TBUT <5 s or Schirmer ≤10 mm with numeric staining thresholds (NEI ≥ 5), the association with HbA1c remained significant (aOR 1.27 per 1%; 95% CI 1.09–1.49; *p* = 0.002, **Table 3**). Restricting the prediabetes stratum to participants meeting both HbA1c and FPG criteria yielded directionally consistent co-primary estimates versus non-diabetes (adjusted ΔNIBUT −1.35 s and adjusted ΔSchirmer −1.70 mm; **Supplementary Table 3**). Separate symptom-only and sign-only endpoint models similarly demonstrated monotonic differences across glycemic strata (**Supplementary Table 3**). Among participants with diabetes, additional adjustment for diabetes duration and treatment regimen did not materially change the association of HbA1c with NIBUT or Schirmer (**Supplementary Table 3**). Results were also similar under alternative eye-level aggregation rules (worse eye for continuous outcomes; both-eye requirement for binary outcomes; **Supplementary Table 3**). Model diagnostics supported linear model assumptions; adjusted *R*^2^ was 0.36 for NIBUT and 0.29 for Schirmer; HbA1c and FPG were correlated (*r* = 0.78), but all variance inflation factors were <2.4. Exclusion of influential observations (Cook’s distance >4/n) changed co-primary *β* estimates by <5%, and logistic models demonstrated acceptable calibration (Hosmer–Lemeshow *p* > 0.20).

## Discussion

In a balanced clinic sample of 300 adults, higher glycemic status was associated with tear-film instability and reduced aqueous secretion, with differences already detectable in prediabetes and persisting after multivariable adjustment. Associations between HbA1c and the co-primary endpoints were approximately linear within the observed range. This extends prior diabetes-versus-non-diabetes syntheses by explicitly including prediabetes within a standardized ocular surface phenotyping framework. Our findings show a coherent, graded pattern in which dysglycemia was associated with tear-film compromise that is biologically plausible within the DED framework. Across strata, NIBUT fell from 11.8 to 10.5 and 9.2 s and Schirmer I from 13.8 to 12.1 and 10.4 mm, with parallel declines in TBUT, higher proportions of osmolarity abnormality (19.0%, 28.0%, 39.0%) and MMP-9 positivity (17.0%, 24.0%, 36.0%), and linear associations per 1% higher HbA1c. These gradients mirror the TFOS DEWS pathophysiology in which evaporative water loss, hyperosmolar stress, and inflammatory amplification are thought to drive loss of homeostasis (18, 19) and align with the clinical use of tear osmolarity as a single marker (20). Systemic oxidative stress and inflammatory activation in dysglycemia have also been documented in meta-analyses, lending biological plausibility to ocular surface inflammatory findings (7, 21). Elevated tear MMP-9 in our more dysglycemic strata is consistent with ocular surface inflammatory activation detected by point-of-care immunoassays and associated with disease severity and treatment response (22, 23). Additionally, a neurotrophic pathway is supported by literature showing corneal small-fiber loss in diabetic peripheral/autonomic neuropathy, which plausibly reduces reflex lacrimation and contributes to the observed Schirmer decrements and NIBUT/TBUT shortening (24–26). Finally, meibomian gland dropout and lipid-layer compromise in type2 diabetes mellitus provide an evaporative mechanism for shorter breakup times that dovetails with our stability findings (27). Together, these inflammatory, neurotrophic, and meibomian-lipid mechanisms substantiate the biological plausibility of the glycemia–DED associations we observed and support using complementary stability (NIBUT/TBUT) and volume (Schirmer/tear meniscus height) endpoints in metabolic eye research. Because neuropathy, cumulative glycemic exposure, and microvascular complications were not systematically phenotyped in the full cohort, these mechanistic interpretations should be regarded as hypothesis-generating rather than directly demonstrated by the present data.

Clinically, the observation that several tear-film and ocular surface metrics differed by glycemic status, including in prediabetes, suggests that ocular surface symptoms and signs may co-occur with early dysglycemia; however, this cross-sectional, quota-sampled clinic study cannot establish temporality or support screening recommendations. The magnitude of between-stratum differences was modest (approximately 1–3 s for NIBUT and 1–3 mm for Schirmer) and should be interpreted in the context of measurement variability and clinically meaningful change thresholds (e.g., DREAM operationalized >2 s for fluorescein TBUT and ≥5 mm for Schirmer; and OSDI has an MCID of ~7–10 points) (28–31). For NIBUT specifically, interpretation at the individual-patient level remains constrained by vest variability (29, 31). However, the group means bracketed the commonly used 10-s threshold for tear-film instability (11.8 s in non-diabetes, 10.5 s in prediabetes, and 9.2 s in diabetes) (12, 19). This threshold-based context, together with directional concordance with TBUT, osmolarity abnormality, and MMP-9 positivity within our dataset, makes the NIBUT finding more clinically interpretable at the group level while still not sufficient for individual-level screening or management decisions. Accordingly, statistical significance at the group level should not be interpreted as establishing clinically meaningful change for individual patients or as sufficient, in isolation, to alter management (28–31). For practical phenotyping, brief chair-time metrics such as NIBUT and Schirmer map to stability and volume, while osmolarity and MMP-9 may provide adjunctive information about homeostatic loss and inflammatory activation (19, 20, 22, 23). More broadly, biomarker panels are increasingly explored for early risk stratification in metabolic disease, supporting the rationale for evaluating adjunctive ocular surface biomarkers alongside glycemic measures (32).

The limitations of this study include the cross-sectional design, which precludes causal inference and cannot establish temporal direction, progression along the glycemic continuum, or whether reverse causation or bidirectional pathways contributed to the observed associations. Accordingly, the observed gradients should be interpreted as between-person associations at a single time point rather than within-person progression. Although we adjusted for key demographic, metabolic, medication, and environmental covariates, residual confounding is possible, including diabetes-related factors not fully captured (e.g., duration of dysglycemia, long-term glycemic exposure, microvascular complications, and autonomic neuropathy). We reported diabetes duration and treatment regimen and performed diabetes-stratum sensitivity models; however, neuropathy and microvascular complications were not systematically phenotyped, limiting adjustment. Thus, the biological pathways discussed above should be interpreted cautiously and as biologically plausible, not as established mediators in this dataset. Recruitment was clinic-based and quota-balanced by glycemic strata, enhancing precision for internal contrasts but limiting external validity; within-sample proportions should not be interpreted as population prevalence, and associations could differ in community-based samples. In addition, quota-based clinic sampling may introduce selection-related distortion of association estimates if factors influencing clinic attendance or successful enrollment were related to both glycemic status and ocular surface measures, even though ocular findings were not used for eligibility. Finally, longitudinal studies are needed to determine whether changes in glycemic control precede changes in ocular surface biomarkers and to establish clinically actionable monitoring thresholds. Future work should include population-based, multicenter longitudinal cohorts to test generalizability and temporality, mechanistic profiling to clarify pathways, and interventional trials in prediabetes and diabetes that evaluate whether improving glycemic control and/or initiating ocular surface therapies can modify NIBUT, Schirmer, inflammatory markers, and patient-reported outcomes, thereby establishing actionable thresholds for treatment along the glycemic continuum.

In this study, we found that higher glycemic status from non-diabetes through prediabetes to diabetes was associated with poorer tear-film stability and lower aqueous secretion, higher osmolarity and MMP-9 positivity, and higher odds of a symptom plus sign DED composite. The co-primary endpoints and adjunctive biomarkers provide complementary phenotyping of ocular surface homeostasis, but longitudinal studies are required to establish temporality and determine the practical relevance of these group-level associations.

## Data Availability

The raw data supporting the conclusions of this article will be made available by the authors, without undue reservation.
