# Supplementary material for: Association of the glucose metabolism continuum (fasting plasma glucose/HbA1c) with tear-film stability and secretion: ocular surface evidence across non-diabetes, prediabetes, and diabetes
Source: Front Endocrinol (Lausanne). 2026 Apr 13;17:1788051. doi: 10.3389/fendo.2026.1788051 (PMC13111086; doi:10.3389/fendo.2026.1788051)
Supplement: Supplementary file 1 [file Table1.docx]

**Supplementary Table S1. Summary of reported analytic families, hypothesis counts, and multiplicity handling**

| **Analytic component** | **Model-level hypotheses (n)** | **Reported p values/contrasts (n)** | **Multiplicity handling/notes** |
| --- | --- | --- | --- |
| Co-primary categorical models (NIBUT and Schirmer by glycemic stratum) | 2 | 6 | Both co-primary endpoints required significant at α=0.05; Pre vs Non, DM vs Non, and p-trend are displayed for each model; no FDR. |
| Secondary categorical models (6 continuous and 4 binary endpoints by glycemic stratum) | 10 | 30 | Benjamini-Hochberg FDR q=0.05 within family. |
| Continuous-exposure main/supportive models (HbA1c and FPG) | 9 | 9 | No FDR for the 2 co-primary HbA1c models; FDR applied to secondary/supportive models where shown. |
| Nonlinearity tests (restricted cubic splines) | 2 | 2 | Exploratory shape assessment; no separate multiplicity adjustment. |
| Sensitivity/robustness models reported numerically | 9 | 17 | Supportive analyses only; includes alternative DED definition, sign-only endpoints, restricted prediabetes, both-eye DED, and diabetes-stratum duration/treatment adjustment. |
| Total | 32 | 64 | Symptom-only OSDI ≥13 was not double-counted because it is identical to the main OSDI ≥13 secondary model. |

**Supplementary Table S2. Prediabetes diagnostic criteria and diabetes clinical characteristics**

| **Characteristic** | **Prediabetes (n=100)** | **Diabetes (n=100)** |
| --- | --- | --- |
| **A. Prediabetes criteria (within prediabetes stratum)** |  |  |
| HbA1c 5.7–6.4% only (FPG <100 mg/dL) | 25 (25.0) |  |
| FPG 100–125 mg/dL only (HbA1c <5.7%) | 15 (15.0) |  |
| Both HbA1c 5.7–6.4% and FPG 100–125 mg/dL | 60 (60.0) |  |
| **B. Diabetes stratum characteristics** |  |  |
| Duration of diagnosed diabetes, years (median [IQR]) |  | 8.0 [3.5, 13.0] |
| Antidiabetic therapy, n (%) |  |  |
| Lifestyle only (no pharmacotherapy) |  | 8 (8.0) |
| Metformin monotherapy |  | 35 (35.0) |
| Other oral agents without insulin |  | 37 (37.0) |
| Insulin-containing regimen (± oral agents) |  | 20 (20.0) |
| Chart-documented complications, n (%) (descriptive only) |  |  |
| Diabetic retinopathy |  | 24 (24.0) |
| Nephropathy/albuminuria |  | 12 (12.0) |
| Peripheral neuropathy |  | 18 (18.0) |

**Supplementary Table S3. Symptom-only, sign-only, and key sensitivity analyses**

| **Analysis** | **Outcome** | **Nondiabetes** | **Prediabetes** | **Diabetes** | **Adjusted effect (95% CI); p-value** |
| --- | --- | --- | --- | --- | --- |
| Symptom-only endpoint | OSDI ≥13, n (%) | 31 (31.0) | 42 (42.0) | 59 (59.0) | Pre vs Non aOR 1.60 (1.00–2.50), p=0.050; DM vs Non aOR 3.20 (1.80–5.70), p<0.001; p-trend<0.001 (q=0.006) |
| Sign-only endpoints (symptoms not required) | NIBUT <10 s, n (%) | 28 (28.0) | 44 (44.0) | 59 (59.0) | Pre vs Non aOR 1.90 (1.10–3.20), p=0.020; DM vs Non aOR 3.60 (2.00–6.60), p<0.001; p-trend<0.001 (q=0.004) |
| Sign-only endpoints (symptoms not required) | Schirmer I ≤10 mm, n (%) | 23 (23.0) | 35 (35.0) | 47 (47.0) | Pre vs Non aOR 1.70 (0.99–2.90), p=0.055; DM vs Non aOR 2.80 (1.60–4.90), p<0.001; p-trend<0.001 (q=0.006) |
| Sign-only endpoints (symptoms not required) | NEI staining ≥2, n (%) | 66 (66.0) | 72 (72.0) | 82 (82.0) | Pre vs Non aOR 1.30 (0.75–2.20), p=0.35; DM vs Non aOR 2.20 (1.20–4.00), p=0.010; p-trend=0.013 (q=0.020) |
| **Sensitivity: Prediabetes restricted to both HbA1c and FPG criteria (n=60)** | | | | | |
| Sensitivity (restricted prediabetes) | NIBUT (s), mean±SD | 11.8 ± 3.1 | 10.3 ± 3.1 (n=60) | 9.2 ± 3.3 | Adjusted Δ vs nondiabetes −1.35 (−2.21 to −0.49), p=0.002 |
| Sensitivity (restricted prediabetes) | Schirmer I (mm), mean±SD | 13.8 ± 5.2 | 11.7 ± 5.3 (n=60) | 10.4 ± 5.6 | Adjusted Δ vs nondiabetes −1.70 (−3.00 to −0.40), p=0.010 |
| Sensitivity: both-eye requirement for DED composite | DED (both eyes meet criteria), n (%) | 12 (12.0) | 18 (18.0) | 28 (28.0) | Pre vs Non aOR 1.55 (0.79–3.04), p=0.20; DM vs Non aOR 2.85 (1.35–6.01), p=0.006; p-trend=0.004 |
| Sensitivity (diabetes stratum only) | HbA1c per 1% to NIBUT & Schirmer |  |  |  | Additional adjustment for diabetes duration and treatment: β_NIBUT −0.65 (−0.95 to −0.35), p<0.001; β_Schirmer −1.05 (−1.55 to −0.55), p<0.001 |
